# Does splitting sleep improve long-term memory in chronically sleep deprived adolescents?

**DOI:** 10.1038/s41539-019-0047-z

**Published:** 2019-06-28

**Authors:** James N. Cousins, Elaine van Rijn, Ju Lynn Ong, Kian F. Wong, Michael W. L. Chee

**Affiliations:** Centre for Cognitive Neuroscience, Duke-NUS Medical School, Singapore, 169857 Singapore

**Keywords:** Human behaviour, Education

## Abstract

Sleep aids the encoding and consolidation of declarative memories, but many adolescents do not obtain the recommended amount of sleep each night. After a normal night of sleep, there is abundant evidence that a daytime nap enhances the consolidation of material learned before sleep and also improves the encoding of new information upon waking. However, it remains unclear how learning is affected when sleep is split between nocturnal and daytime nap periods during a typical school week of restricted sleep. We compared long-term memory in 58 adolescents who underwent two simulated school weeks of suboptimal continuous (6.5 h nocturnal sleep opportunity) or split sleep (5 h nocturnal sleep +1.5 h daytime nap at 14:00). In the first week, participants encoded pictures in the late afternoon on Day 5 and were tested after 2-nights of recovery sleep. On 3 consecutive days in the second week, participants learned about six species of amphibians in the morning, and six different amphibians in the late afternoon. Testing was performed in the evening following a night of recovery sleep. In the first week, the split sleep group recognized more pictures. In the second week, they remembered more facts about species learned in the afternoon. Groups did not differ for species learned in the morning. This suggests that under conditions of sleep restriction, a split sleep schedule benefits learning after a nap opportunity without impairing morning learning, despite less preceding nocturnal sleep. While not replacing adequate nocturnal sleep, a split sleep schedule may be beneficial for chronically sleep restricted learners.

## Introduction

Sleep after learning stabilizes and integrates memories for long-term storage,^1^ while sleep prior to learning prepares the brain to encode new information.^2^ However, many adolescents obtain insufficient sleep, with 68% of teenagers in the US reporting sleep below the recommended 8–10 h each night^3,4^ Actigraphically assessed sleep length has been consistently shown to dip below 6 h on weekday nights for adolescents in East Asia.^5,6^ Chronic sleep restriction is associated with poorer academic performance^7^ and a reduced capacity to encode new information.^6^ Delaying school start times^8^ and setting limits on bedtime^9,10^ can improve adolescent sleep, but implementing these will entail significant effort and take time to employ effectively. In the interim, it remains an open challenge to improve learning given the restricted sleep adolescents currently obtain.

Daytime naps consistently enhance memory in laboratory studies involving children^11–13^ and adults.^14–18^ Recently these findings have been translated to classroom learning by examining the utility of napping using educationally realistic learning materials. When learning detailed facts about ecology, a mid-afternoon nap enhanced memory to a similar extent as when that time was spent cramming.^19^ A separate study found that a 2 h nap opportunity after a lecture was associated with better test performance in adolescents when compared to those who attended regular classes,^20^ while a third study found a similar advantage when weekly lessons were followed by naps of longer than 30 min.^21^

These findings suggest that it might be preferable to split available sleep time between a nocturnal bout and a daytime nap. Critically however, prior experimental research exploring the memory benefits of napping has only examined napping as a supplement to a fixed amount of nocturnal sleep.^11–15,17–19^ As such, nap conditions were associated with more total sleep in the 24 h period prior to cognitive tests compared to wake conditions that did not include a nap. Enhanced post-nap cognition could therefore be a function of total sleep obtained, rather than the nap per se. Determining if naps can benefit learning and memory when total sleep duration is held constant is crucial, as it could inform whether educational institutions should make the considerable practical adjustments that would facilitate napping in schools, or whether alternate solutions that focus on nocturnal sleep would provide similar benefits.

A handful of studies—conducted in adults with a view to optimize shift-work—controlled for total sleep obtained by splitting sleep into nocturnal and afternoon nap periods,^22^ or two equivalent periods across 24 h.^23,24^ Performance on a battery of cognitive tests was compared with continuous nocturnal sleep of the same overall duration. Measures of subjective alertness, psychomotor vigilance and processing speed were found to be determined by total sleep obtained, rather than how that sleep was distributed across the day.^22–24^ It remains to be determined how long-term memory is affected when learning takes place under a similar split sleep schedule.

**Fig. 1:**
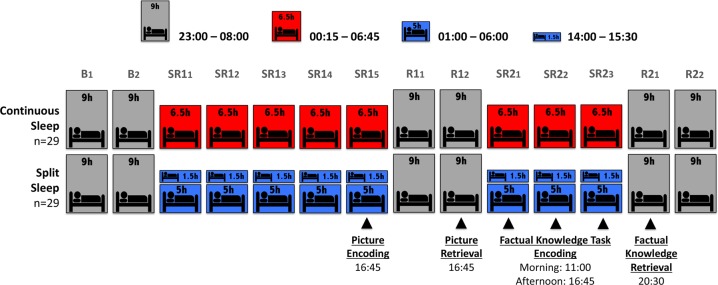
Study Protocol. The study began with 2 baseline nights (B_1_–B_2_) of 9 h nocturnal sleep opportunity followed by the first school week period, during which one group were provided with a 6.5 h nocturnal sleep opportunity (00:15–06:45) and the other group 5 h nocturnal sleep opportunity (01:00–06:00) with a 1.5 h afternoon nap (14:00–15:30). This was followed by a “weekend” recovery period of 9 h nocturnal sleep opportunity (R1_1_–R1_2_). The second weekday (SR2_1_–SR2_3_) and weekend recovery period (R2_1_–R2_2_) of the experiment matched the first, except with 3-days of manipulation rather than 5. Picture encoding began at the end of the first manipulation period (SR1_5_) and was tested after two nights of recovery (R1_2_). Learning of factual knowledge took place in the morning and afternoon on each weekday of the second manipulation period (SR2_1_–SR2_3_) and was tested after one night of weekend recovery sleep (R2_1_)

**Fig. 2:**
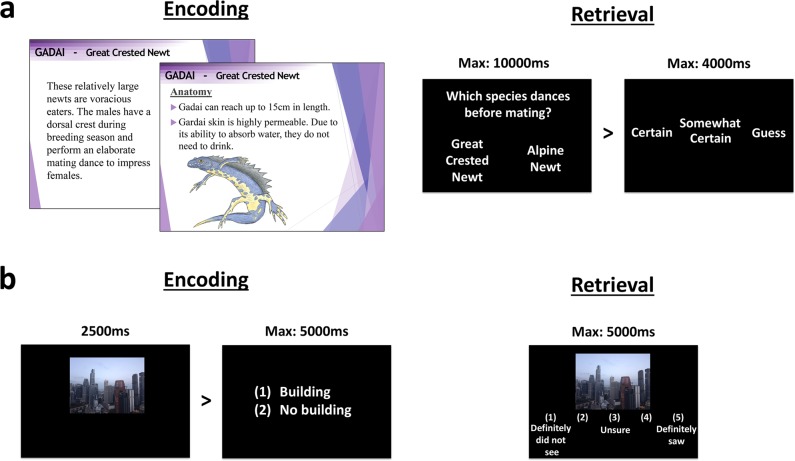
Stimuli. **a** Factual knowledge encoding consisted of detailed information about 12 species of amphibians presented on slides. The test probed 360 2-alternative forced choice questions of varying difficulty, each followed by a confidence rating. **b** For the picture encoding task, participants viewed 160 images each followed by a building/no-building judgment. During retrieval, 160 old images were presented with an additional 80 new images, and participants indicated their confidence that it was an old or new image on a 5-point scale

To address these gaps in our knowledge, we compared long-term memory in adolescents who learned across two simulated weeks of restricted sleep in a naturalistic school setting, where sleep opportunities on each day were either split into 5 h nocturnal sleep and a 1.5 h daytime nap, or 6.5 h of continuous nocturnal sleep (Fig. 1). During the second week and for three consecutive days, participants spent an hour learning detailed facts about six amphibians in the morning, and another hour learning about six different amphibians after the nap in the late afternoon (Factual Knowledge Task; Fig. 2a). Participants were then tested the following evening after recovery sleep (9 h time-in-bed (TIB)). This makes provision for both groups being rested during retrieval, so that observed memory effects could be reasonably assigned to the effects of splitting sleep on prior encoding and/or consolidation, rather than retrieval. We determined whether memory for material learned in the morning and afternoon would differ between groups, since there are different encoding and consolidation advantages for each. Specifically, the relative loss of nocturnal sleep in the split sleep group could impair their ability to encode in the morning.^6,25,26^ However, the subsequent nap might compensate by enhancing consolidation of what was learned.^18^ If memory were purely a function of total sleep obtained, then memory for information learned in the morning or afternoon would not differ between split and continuous sleep schedules. This design permits the evaluation of how splitting sleep affects learning that is similar to the way students study and revise across multiple episodes in school. However, this methodology does not allow a clear attribution of group differences to encoding or consolidation.

To assess the effect of splitting sleep specifically on encoding, participants performed a separate task during the first week of the protocol: encoding pictures^6^ in the late afternoon on the fifth day of sleep restriction. Recognition of those pictures was tested after two nights of recovery sleep (9 h TIB; Fig. 2b). Since the opportunity to consolidate during this retention interval was the same in the two sleep conditions, performance at retrieval provides a reliable measure of encoding success.^6,25^ Prior work has shown improved encoding after a single daytime nap,^17^ motivating us to test if encoding capacity would also be enhanced under a habitual napping schedule where prior sleep was matched between nap and no-nap conditions.

## Results

### Actigraphy

Term-time sleep habits were actigraphically assessed for a one-week period occurring within 5 months prior to commencement of the study. Participants demonstrated a common pattern of shortened sleep on weekdays (TIB = 6.83 ± 0.94, total-sleep-time (TST) = 5.44 ± 0.84) and extension of sleep on weekends (TIB = 8.31 ± 1.00, TST = 6.69 ± 0.96; Table 1). Participants also adhered to a sleep schedule (23:00–08:00) in the week prior to commencement of the study, confirmed with actigraphy (TIB = 8.9 ± 0.37, TST = 7.45 ± 0.53). Actigraphy and polysomnography during the study indicated that TST was effectively altered in line with our experiment design during each night of the experiment.

**Table 1:** Screening characteristics

	Continuous sleep		Split sleep		*t*/*χ*^2^	*p*
	Mean	SD	Mean	SD		
*n*	29	–	29	–	–	–
Age (years)	16.58	1.12	16.55	0.74	0.12	0.907
Gender (number of males)	15	–	15	–	0.04	0.998
Caffeinated drinks per day	0.58	0.80	0.55	0.69	0.17	0.868
Body mass index	21.25	3.46	20.67	2.80	0.69	0.491
Raven’s advanced progressive matrices score	8.83	1.91	9.21	1.63	−0.81	0.420
Beck anxiety inventory score	9.34	6.68	10.38	6.30	−0.61	0.546
Beck depression inventory score	10.97	5.29	9.21	5.53	1.24	0.221
Morningness-eveningness questionnaire score	48.97	7.54	50.72	7.07	−0.92	0.364
Epworth sleepiness scale score	8.21	3.43	7.86	3.78	0.36	0.717
Chronic sleep reduction questionnaire						
Total score	35.24	5.96	36.10	4.66	−0.61	0.542
Shortness of sleep	12.72	2.09	13.03	2.04	−0.57	0.569
Irritation	6.38	1.52	6.76	1.90	−0.84	0.405
Loss of energy	8.48	2.05	8.03	2.01	0.84	0.403
Sleepiness	7.66	2.27	8.28	1.51	−1.23	0.226
Pittsburgh sleep quality index global score	4.48	1.50	4.17	1.77	0.72	0.475
Actigraphy						
TIB on weekdays (h)	7.00	0.77	6.84	1.13	0.63	0.530
TIB on weekends (h)	8.45	1.13	8.15	1.05	1.07	0.291
TIB on average (h)	7.42	0.63	7.22	0.91	0.98	0.329
TST on weekdays (h)	5.51	0.75	5.50	0.89	0.02	0.988
TST on weekends (h)	6.76	1.14	6.64	1.00	0.42	0.674
TST on average (h)	5.86	0.68	5.83	0.73	0.20	0.845
Sleep efficiency (%)	79.02	5.57	81.04	6.64	−1.26	0.215

*y* year, *SD* standard deviation, *TIB* time in bed, *TST* total sleep time, *h* hour, actigraphy threshold: medium

### Memory

#### Picture encoding

**Fig. 3:**
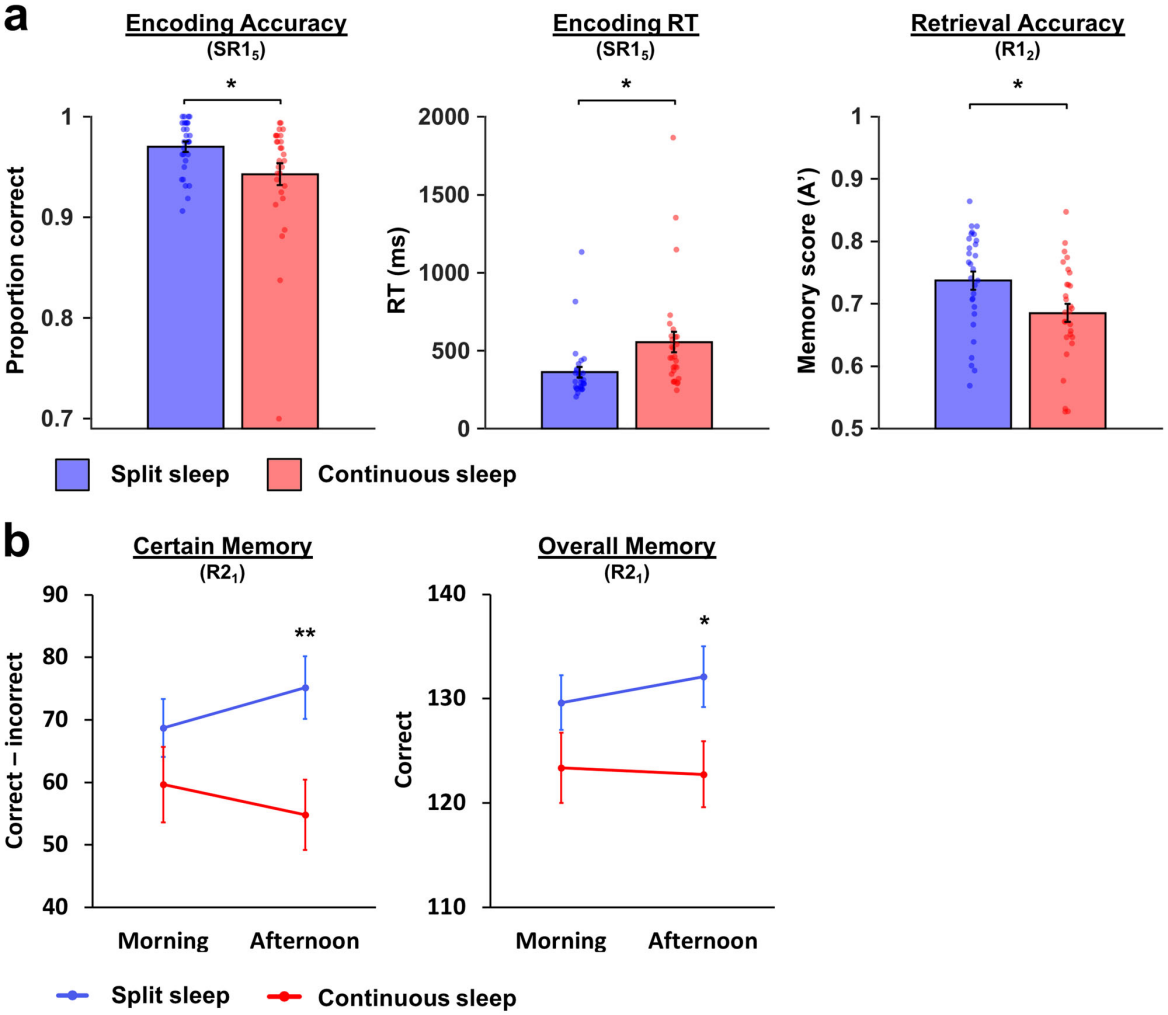
Memory performance. **a** For picture encoding, accuracy was higher and response times were faster for the split sleep group during the building/no-building judgments at encoding (SR1_5_). Picture recognition scores (A′) were also significantly higher for the split sleep group. **b** Memory in the factual knowledge task for species learned in the afternoon was significantly better in the split sleep group, while morning learning was not significantly different between groups. Mean ± standard error of the mean (SEM). *p < 0.05, **p < 0.01

Encoding took place after five days on the split/continuous sleep schedule (14:45 on day SR1_5_). Participants incidentally encoded pictures of landscapes by making building/no-building judgments and were not informed that memory for images would be tested later. Both groups were highly accurate at judging pictures to contain a building or not (6.5 h = 0.94 ± 0.06; split sleep = 0.97 ± 0.03) and significantly higher in the split sleep group, t(56) = 2.241, *p* = 0.029 (Fig. 3a).

During retrieval 48 h later (16:45 on day R1_2_), all participants indicated that they were unaware they would be tested for their memory of the previous session. Recognition of previously viewed images was tested alongside new images. The signal detection measure A′ was utilized to assess memory as it corrects for response bias. A′ scores were significantly higher in the split sleep group, t(56) = 2.505, *p* = 0.015, Cohen’s *d* = 0.66 (Table 2). These findings indicate a clear afternoon encoding advantage associated with a split sleep schedule.

**Table 2:** Retrieval for picture encoding and the factual knowledge task

	Continuous sleep		Split sleep	
	Mean	SD	Mean	SD
Picture encoding				
A′	0.69	0.08	0.74	0.08*
Hits (proportion)	0.38	0.13	0.45	0.13
False alarms (proportion)	0.20	0.12	0.17	0.10
Misses (proportion)	0.39	0.18	0.44	0.15
Correct rejections (proportion)	0.56	0.24	0.70	0.18*
Factual knowledge task				
Overall memory				
Morning correct	123.38	18.07	129.62	14.10
Afternoon correct	122.76	17.04	132.10	15.62*
Certain				
Morning correct	76.72	35.44	82.59	26.13
Morning incorrect	17.07	16.06	13.86	10.65
Morning (correct–incorrect)	59.65	32.64	68.73	24.93
Afternoon correct	72.52	33.38	87.45	25.60
Afternoon incorrect	17.69	17.82	12.28	9.19
Afternoon (correct–incorrect)	54.83	30.18	75.17	27.14**
Somewhat certain				
Morning correct	24.86	14.66	24.21	12.66
Morning incorrect	16.24	10.59	12.66	9.36
Morning (correct–incorrect)	8.62	10.03	11.55	9.64
Afternoon correct	26.07	14.64	22.79	14.17
Afternoon incorrect	16.52	11.72	13.66	8.99
Afternoon (correct–incorrect)	9.55	10.51	9.13	11.20
Guess				
Morning correct	21.24	17.50	22.41	14.42
Morning incorrect	21.59	16.47	22.31	14.20
Morning (correct–incorrect)	−0.35	8.45	0.10	7.19
Afternoon correct	23.83	20.57	21.45	14.54
Afternoon incorrect	21.48	16.32	20.83	10.94
Afternoon (correct–incorrect)	2.35	9.00	0.62	7.81

Factual knowledge task: 360 questions split between morning (180) and afternoon (180)

*SD* standard deviation

*p < 0.05, **p < 0.01

#### Factual knowledge task—pretest

Participants performed a pretest prior to learning to establish their existing knowledge about amphibians. Two-alternative forced choice questions tested general facts about amphibians (general knowledge), specific facts about the species they were about to learn (specific knowledge), and ability to identify species from images (picture identification). Participants were also asked to rate their subjective level of knowledge and disgust (1 = low, 9 = high) when presented with a picture of each species. There were no significant group differences for general knowledge, *t*(56) = 1.395, *p* = 0.168, specific knowledge, *t*(56) = 0.579, *p* = 0.565, or picture identification *t*(56) = 0.349, *p* = 0.728. General knowledge about amphibians was relatively low (*M* = 61.55 ± 10.44% correct, chance level 50%), but significantly above chance (*p* < 0.001). Specific knowledge (*M* = 49.66 ± 9.03%) and picture identification (*M* = 52.88 ± 12.31%) did not differ from chance (*p* > 0.05).

There was a non-significant trend for subjective knowledge to be higher in the continuous sleep group, *t*(56) = 1.843, *p* = 0.071, but ratings in this group were still very low considering a score of 1 represented “no knowledge” (*M* = 1.42 ± 0.64). On average participants rated all species as moderately disgusting (*M* = 4.69 ± 2.32), but these ratings did not differ between groups, *t*(56) = 1.069, *p* = 0.289.

#### Factual knowledge task—retrieval

Retrieval was tested at 20:30 on day SR2_1_ via two-alternative forced choice questions, followed by confidence ratings (certain, somewhat certain and guess). Responses within each rating were corrected for response bias (correct–incorrect). Any group differences were expected to be present for participant’s most confident memories (certain responses),^19^ which are the least prone to noise introduced by guessing. For certain memories, a mixed ANOVA with group (split sleep/continuous sleep) and time (morning/afternoon) showed a significant main effect of group, *F*(1,56) = 4.172, *p* = 0.046, an interaction, *F*(1,56) = 5.646, *p* = 0.021, but no effect of time, *F*(1,56) = 0.117, *p* = 0.734 (Fig. 3b). Independent samples *t*-tests revealed significantly better memory of species learned in the afternoon for the split sleep group relative to the continuous sleep group, *t*(56) = 2.7, *p* = 0.009, Cohen’s *d* *=* 0.71, while groups did not differ significantly for morning learning, *t*(56) = 1.189, *p* = 0.239, Cohen’s *d* *=* 0.31. Within the split sleep group, the higher performance for afternoon compared to morning learning was significant, *t*(28) = 2.088, *p* = 0.046, while the numerically lower performance for afternoon compared to morning learning observed in the continuous sleep group was not, *t*(28) = 1.34, *p* = 0.191. This indicates that a split sleep schedule improves afternoon learning without any cost to morning learning.

Overall memory showed a trend for a main group effect, *F*(1,56) = 3.825, *p* = 0.055, but no effect of time, *F*(1,56) = 0.363, *p* = 0.549, and no interaction, *F*(1,56) = 1.008, *p* = 0.32. Planned comparisons showed a similar pattern to certain memory, with a significantly better memory for afternoon learning in the split sleep group, *t*(56) = 2.177, *p* = 0.034, Cohen’s *d* *=* 0.57, but no group difference for morning learning, *t*(56) = 1.467, *p* = 0.148, Cohen’s *d* *=* 0.39, and no within group differences between morning and afternoon learning (*p* > 0.19).

There were no significant main effects or interactions for somewhat certain (group: *F*(1,56) = 0.273, *p* = 0.603, time: *F*(1,56) = 0.343, *p* = 0.561, group * time interaction: *F*(1,56) = 1.743, *p* = 0.192), or guess responses (group: *F*(1,56) = 0.15, *p* = 0.7, time: *F*(1,56) = 1.388, *p* = 0.244, group * time interaction: *F*(1,56) = 0.637, *p* = 0.428).

#### Factual knowledge task—subjective measures

**Fig. 4:**
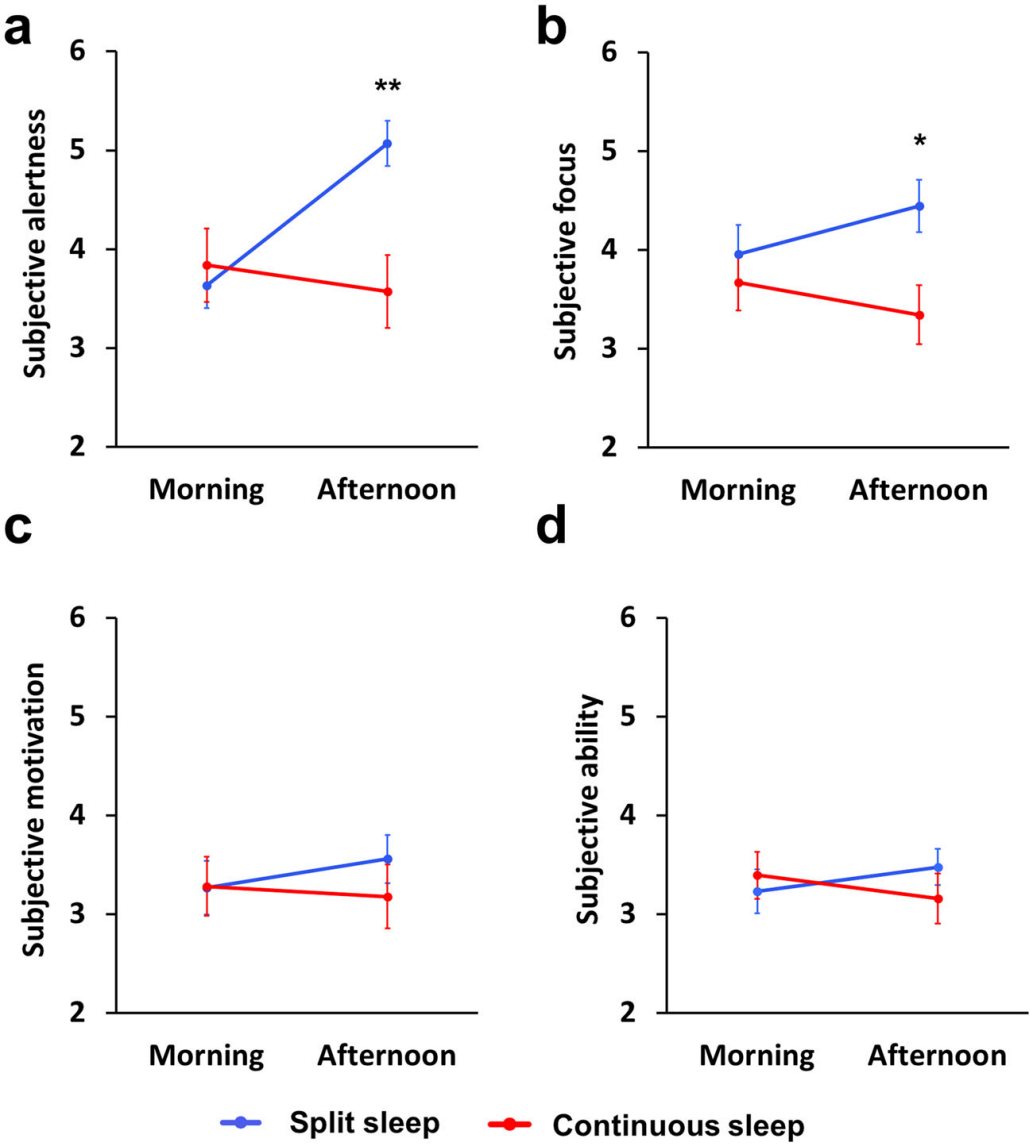
Subjective measures during factual knowledge learning. The split sleep group had significantly greater subjective **a** alertness and **b** focus in the afternoon, while subjective **c** motivation and **d** ability to learn did not differ between groups. *p < 0.05, **p < 0.01

We explored whether a split sleep schedule impacted on participant’s subjective impressions of their learning capabilities while they were studying the material. We asked four questions that probed subjective motivation, focus on the task, ability to learn, and alertness (Karolinska Sleepiness Scale: KSS). Morning and afternoon sessions consisted of two 30 min blocks separated by a 2 min break. Questions were administered during the break and at the end of the block. Ratings were collapsed across all three days to create separate means for morning and afternoon sessions (Fig. 4). ANOVA including group (split sleep, continuous sleep) and time (morning, afternoon) identified no main effects for focus, ability, or motivation (*p* > 0.05), but there were significant time * group interactions for focus, *F*(1,56) = 7.724, *p* = 0.007, and ability, *F*(1,56) = 5.976, *p* = 0.018. Consistent with the memory findings, focus on the task was significantly higher in the split sleep group in the afternoon, *t*(56) = 2.647, *p* = 0.011, but groups did not differ in the morning session, *t*(56) = 0.894, *p* = 0.375. The interaction for ability showed a similar pattern (Fig. 4b), but direct group comparisons did not differ in morning or afternoon sessions (*p* > 0.05). Subjective alertness showed a significant main effect of time, *F*(1,56) = 13.072, *p* = 0.001, and a group * time interaction *F*(1,56) = 25.62. *p* < 0.001, but no group effect, *F*(1,56) = 2.6, *p* = 0.112. Alertness was significantly higher in the afternoon for the split sleep group, *t*(56) = 3.34, *p* = 0.001, but groups did not differ in the morning, *t*(56) = 0.291, *p* = 0.772.

Last, we correlated these subjective measures with certain memory scores within the appropriate time period (e.g., morning alertness with memory for species learned in the morning) within each group separately, making 16 comparisons in total (false discovery rate corrected).^27^ Significant relationships were only observed for the split sleep group, where memory for morning species was positively correlated with focus (*R*_s_ = 0.52, *p* = 0.004) and ability (*R*_s_ = 0.532, *p* = 0.003), while afternoon memory correlated significantly with ability (*R*_s_ = 0.519, *p* = 0.004).

To summarize, the split sleep schedule led to higher levels of subjective alertness and focus in the afternoon after the nap, although correlations between these subjective measures and afternoon memory were not significant.

#### Psychomotor vigilance and memory task response times

Psychomotor vigilance was assessed shortly before the learning (10:15 and 16:30) and test sessions for picture encoding (16:30) and the factual knowledge task (20:15). Response times during both tasks were also analyzed as indirect measures of alertness, although note that participants were instructed to respond as accurately as possible for both memory tasks without any mention of the speed of responses.

Prior to picture encoding (16:30 on SR1_5_), the continuous sleep group had significantly more lapses, *t*(56) = 4.806, *p* < 0.001. Response times during the encoding task were also significantly slower in the continuous sleep group, *t*(56) = 2.615, *p* = 0.011, providing a congruent indication of reduced vigilance/sustained attention during the encoding session. Prior to picture retrieval (16:30 on R1_2_), the continuous sleep group still had significantly more lapses, *t*(56) = 2.607, *p* = 0.012, but this time they were faster to respond than the split sleep group during the picture retrieval session at 16:45, *t*(56) = 4.387, *p* < 0.001. The higher number of lapses for the continuous sleep group prior to picture retrieval raises the possibility that observed memory impairments are related to lapses of attention during retrieval, rather than a deficit to prior encoding. However, PVT lapses (16:30 on R1_2_) did not significantly correlate with memory (A′) in either experimental group (*p* > 0.05).

In the second week of the experiment, lapses for PVT’s occurring prior to the factual knowledge task in the morning (10:15) and afternoon (16:30) were collapsed across the 3 learning days (SR2_1_ to SR2_3_) and analyzed via a mixed ANOVA that included the factors group and time. This showed a significant main effect of time, *F*(1,56) = 9.795, *p* = 0.003, group, *F*(1,56) = 8.465, *p* = 0.005, and a group * time interaction, *F*(1,56) = 25.3, *p* < 0.001. Groups did not differ in the morning, *t*(56) = 0.851, *p* = 0.398, although the split sleep group had significantly fewer lapses than the continuous sleep group in the afternoon *t*(56) = 4.987, *p* < 0.001.

There were no group differences for the PVT performed before the factual knowledge retrieval test at 20:15 on R2_1_, *t*(56) = 1.269, *p* = 0.21. Similar to the picture encoding task, response times during retrieval were significantly faster in the continuous sleep group, *t*(56) = 2.147, *p* = 0.036.

### Polysomnography

**Fig. 5:**
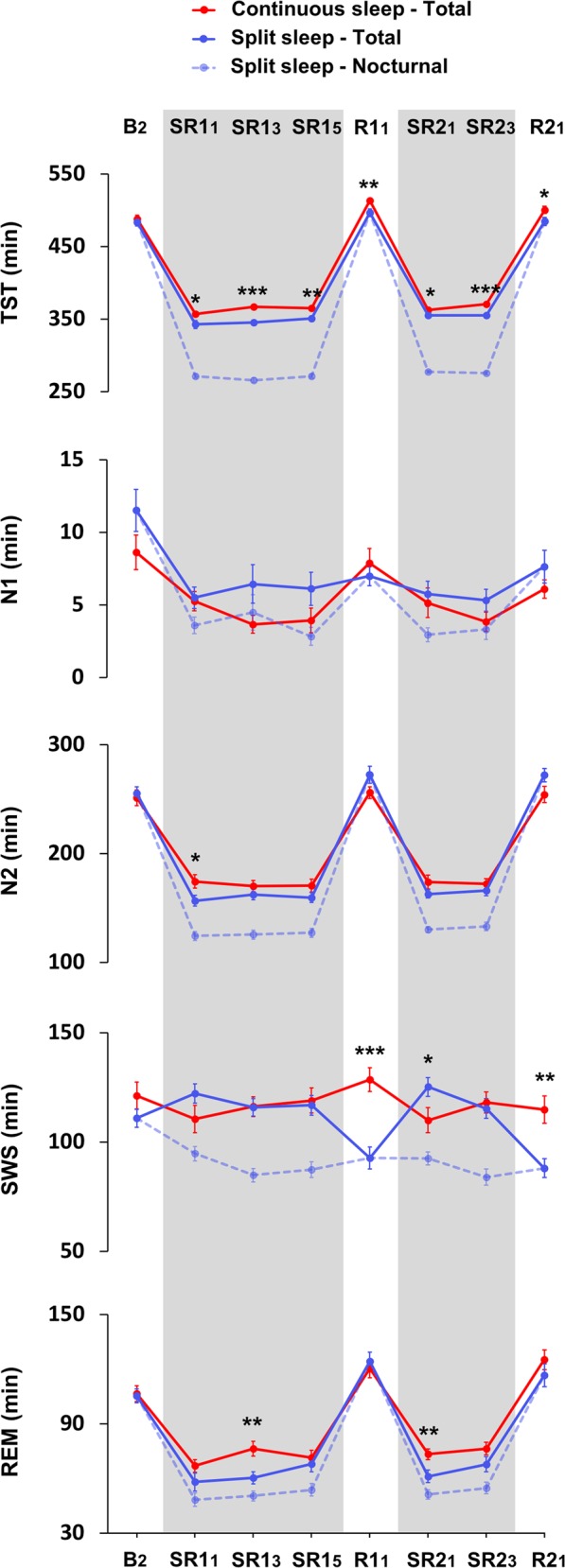
Sleep macrostructure as assessed with polysomnography. The split sleep schedule was associated with significantly reduced TST across most manipulation days. The underlying macrostructure was also affected, although there were no consistent reductions in any specific sleep stage during sleep restriction. ***p < 0.001, **p < 0.01, *p < 0.05 for significant group contrasts comparing sleep obtained across each 24 h period for the continuous sleep group (solid red) and split sleep group (solid blue). Dashed blue line indicates nocturnal sleep only for the split sleep group. Gray shaded areas mark sleep restriction periods (SR)

When considering total sleep during each 24 h period (i.e., nocturnal and nap sleep combined for the split sleep group), the split sleep schedule was associated with consistently reduced TST (Fig. 5). There were two consistent changes in sleep architecture throughout the protocol. First, rapid eye movement (REM) sleep for the split sleep group was significantly reduced on two separate days in the first (SR1_3_) and second week (SR2_1_) (*p* < 0.01). Second, the continuous sleep group obtained significantly more slow wave sleep (SWS) on recovery days R1_1_ and R2_1_ (*p* < 0.01), indicating greater accumulation of sleep pressure in participants who did not nap in the afternoon prior to recovery sleep (see Supplementary Table 1 for nap sleep macrostructure). Group comparison of only nocturnal sleep showed the expected decrease in TST on all SR days for the split sleep group, and significantly reduced time spent in all sleep stages (*p* < 0.05) except for N1 (*p* > 0.05).

Subsequent analyses focused on sleep stage duration during sleep periods within 24 h of each task. For picture encoding, nocturnal and afternoon nap sleep times were combined (SR1_5_). Although groups did not significantly differ in the length of any individual sleep stage (*p* > 0.05), the split sleep group had significantly lower total sleep time, *t*(53) = 3.173, *p* = 0.003, by approximately 15 min (split sleep: *M* = 350.52 ± 19.36 min; continuous sleep: *M* = 365.24 ± 14.64 min). Certain memory did not significantly correlate with any sleep stage duration or TST (*p* > 0.05).

Since learning in the factual knowledge task took place over 3 days (SR2_1_–SR2_3_), we examined averaged sleep characteristics across the two days where PSG was recorded (SR2_1_ and SR2_3_; Fig. 5). For combined night and daytime sleep, TST was again significantly lower in the split sleep group (split sleep: *M* = 354.71 ± 9.57 min; continuous sleep: *M* = 366.69 ± 9.41 min), *t*(53) = 4.682, *p* < 0.001. Analysis of macrostructure showed that REM sleep duration was significantly reduced by splitting sleep (split sleep: *M* = 64.0 ± 17.38 min; continuous sleep: *M* = 75.36 ± 13.55 min), *t*(53) = 2.697, *p* = 0.009, while other sleep stages did not differ (*p* > 0.05). Again, certain memory in the morning or afternoon did not correlate significantly with any of these sleep metrics (*p* > 0.05).

## Discussion

We assessed the incidental encoding of pictures and learning of educationally realistic factual knowledge in sleep restricted adolescents under two sleep schedules: split sleep and continuous sleep. We found that splitting sleep between a nocturnal period (5 h sleep opportunity) and a short daytime nap (1.5 h sleep opportunity) resulted in better recall of learned material compared to a purely nocturnal schedule with the same total sleep (6.5 h), specifically for information learned after the nap in the afternoon. Critically, the split sleep schedule did not impair memory for information learned in the morning, despite less nocturnal sleep and less total sleep across 24 h. These findings expand upon prior observations that a daytime nap benefits long-term memory,^17–21^ by showing that improvements remain under a habitual napping schedule when total available sleep time is matched between nap and no-nap conditions.

The optimization of sleep schedules has been proposed as a low cost way to improve educational outcomes.^28^ The afternoon learning improvement we observed for both memory tasks suggests that splitting sleep could be one such strategy to improve learning. The 90 min mid-afternoon sleep opportunity in the current study was chosen to maximize the probability of participants obtaining a full cycle of N2, SWS and REM sleep,^29^ but is toward the upper limits of nap durations that may be practical to implement in schools. Relatively short naps of 30–60 min have been shown to provide benefits to memory lasting up to a week,^15,19,21^ therefore future studies should examine whether splitting sleep with shorter nap opportunities provides similar benefits for cognition.

Most prior work has examined the benefit of naps in relation to the consolidation of material learned before sleep, but there are several indications that the nap advantage we observed is the result of enhanced encoding, rather than consolidation. The picture encoding task provides a relatively pure measure of encoding capacity, because the incidental nature of the task restricts the use of mnemonic strategies, while only those trials when participants are paying attention and make the correct building/no-building judgment were analyzed. The improved afternoon encoding we observed for the split sleep group on this task also indirectly suggests that improved afternoon learning for the factual knowledge task was related to enhanced encoding, rather than consolidation. This conclusion also seems likely given that the split sleep group obtained less nocturnal sleep in which to consolidate factual knowledge learned in the afternoon.

As splitting sleep resulted in less TST over 24 h, it was surprising that this group had an afternoon memory advantage without any negative impact on information learned in the morning. Although the mechanism underlying this observation cannot be directly explained with the current design—where learning was spread over several days and learning sessions—we posit that the synaptic homeostasis hypothesis^2^ provides a plausible mechanistic account. Synaptic connections are potentiated during wakefulness, and slow-wave activity is proposed to globally downscale net synaptic weight to avoid saturation in memory networks. This is proposed to facilitate new learning. Although the split sleep group obtained significantly less total SWS in the night prior to morning learning, most SWS takes place in the first 4 h of nocturnal sleep. Here, both groups obtained this early period of slow-wave rich sleep, which may be sufficient to restore encoding capacity. This hypothesis is supported by a pair of studies that show encoding to be comparable between those obtaining half a night (~4 h) and a full night of sleep (~8 h).^30,31^ It is well established that total^25,32^ or chronic sleep deprivation^6^ impairs encoding, but the relatively small difference between 5 and 6.5 h nocturnal sleep appears not to adversely affect encoding. The afternoon nap however, provides an additional period of downscaling for the split sleep group, and this may account for the difference in afternoon encoding capacity we observed.

It is important to note that while splitting sleep had no adverse effects for declarative memory, several other cognitive faculties linked to REM sleep may have been affected, including procedural memory,^33,34^ creative problem solving^35^, and emotional regulation.^36^ With regard to the latter, recently published data from the current study protocol showed that the split sleep schedule was associated with improved positive mood,^37^ despite reductions in total time spent in REM sleep. The split sleep schedule could be optimized to facilitate REM sleep, for example by slightly extending morning sleep, or taking naps earlier in the morning. Further studies are needed to optimize sleep schedules, establishing a balance between the cognitive benefits associated with these different aspects of sleep and the practicality of translating this behavior to schools.

Another factor to consider is the circadian variation in cognitive performance, particularly in relation to the afternoon ‘circadian dip’, which may have impacted on both memory tasks. The circadian dip is associated with reduced afternoon levels of alertness^38^ and perhaps also encoding capacity,^17^ therefore splitting sleep may have minimized the impairment associated with learning at this relatively disadvantageous time of day. This was hinted at by a non-significant reduction in memory performance across the day in the continuous sleep group.

The encoding of any stimulus relies on a complex interaction of factors that includes focusing and sustaining attention on a stimulus, as well as cognitive control processes that allocate resources to the goal of forming an enduring memory trace. These underlying cognitive processes deteriorate with sleep loss^39^ and are enhanced by daytime naps,^40,41^ therefore these factors may have contributed to the afternoon learning advantage for the split sleep group, particularly for the long and demanding learning blocks of the factual knowledge task. Consistent with this, measures of post-nap psychomotor vigilance, subjective alertness and subjective ability to focus during learning blocks of this task were significantly higher in the split sleep group, therefore it is likely that this contributed to their improved learning capabilities.

A potential limitation to the picture encoding task is that vigilance in the continuous sleep group prior to the retrieval session remained significantly lower than the split sleep group. Thus, impaired memory of the continuous sleep group may be due to decreased vigilance during retrieval, despite the two nights of intervening recovery sleep intended to equalize vigilance across groups. However, attentional lapses did not significantly correlate with memory performance. Additionally, the few studies that have assessed the effect of sleep loss specifically on retrieval have found no significant impairments after a night of total sleep deprivation,^42–44^ even though this level of sleep loss produces a consistent vigilance impairment.^39^ The group differences we observed in vigilance therefore seem unlikely to account for memory impairments, although we cannot entirely rule out this possibility. For the factual knowledge task, vigilance did not differ between groups prior to retrieval. Moreover, we observed an interaction between memory for information learned in the morning and afternoon: if vigilance during retrieval was an influential factor then memory for all information should be equally affected, regardless of when it was learned.

A significant limitation to broad generalization of our work may be that the sleep length in our split and continuous groups was below the 8–10 h recommended for adolescents.^3^ In the absence of a control group obtaining the recommended amount of sleep, we cannot determine the level of impairment associated with a 6.5 h TIB sleep schedule relative to the recommended sleep duration, or whether splitting sleep is also advantageous for individuals with habitual sleep length that is closer to the recommended amount. However, this sleep schedule provides an accurate reflection of sleep obtained by adolescents undergoing habitual chronic sleep restriction in East Asia,^5,6^ and around the world,^45^ therefore our findings suggest that facilitation of naps in schools would be beneficial for memory in a range of adolescent populations.

Another factor to consider for the potential translation of these findings to schools is the spacing of learning and how that relates to sleep. Spaced learning results in superior retention of memoranda although the underlying reasons are debated.^46^ Spaced learning was recently shown to limit the deleterious impact of multi-night sleep restriction on vocabulary learning.^47^ As factual knowledge learning in both conditions of the current study was spaced, further work will be needed to assess whether the interaction between spacing and sleep contribute to the observed effects.

To conclude, while adequate nocturnal sleep should continue to be strongly advised, under conditions of sleep restriction, a split sleep schedule appears to be preferable for harnessing the cognitive functions that underpin learning in the afternoon, without negatively impacting upon morning learning. These findings contribute towards growing experimental evidence that facilitating naps in schools may be an effective measure to improve learning outcomes^20,21^ in chronically sleep deprived adolescents.

## Methods

### Participants

Sixty adolescents (15–19 years of age) were invited to participate in the study from schools throughout Singapore. Participants were screened for known health conditions, sleep disorders, and had body mass index of ≤30 kg/m^2^. We avoided recruiting habitual short sleepers (actigraphically assessed TIB < 6 h averaged across weekdays and weekends, with weekend sleep extension ≤1 h). Participants must not have traveled across >2 time zones 1 month prior to the study, and not consumed ≥5 caffeinated beverages per day.

Two participants withdrew from the study and those who remained (29 male, mean age = 16.6 ± 0.9) were matched into 2 groups: split sleep (*n* = 29) and continuous sleep (*n* = 29). The groups did not differ significantly in age, gender, consumption of caffeinated beverages, non-verbal intelligence, morning-eveningness preference, symptoms of chronic sleep reduction, levels of daytime sleepiness, subjective sleep quality, self-reported and actigraphically assessed sleep habits, or levels of anxiety and depression (*p* > 0.22; Table 1).

The study was approved by the National University of Singapore Institutional Review Board and was conducted in accordance with the ethical standards of the 1964 Helsinki declaration and its later amendments. The study was a registered clinical trial (https://clinicaltrials.gov/ct2/show/NCT03333512). All participants and legal guardians provided written informed consent.

### Picture-encoding task

Stimuli were identical to those used in our prior study,^6^ consisting of 240 images depicting a variety of landscapes and building types, of which half featured buildings while the other half did not. These were split into three sets of 80 images (40 buildings, 40 no buildings). Two sets were presented during both encoding and retrieval (160 old images), while one set was presented only at retrieval (80 new images). The use of each set during encoding and retrieval was counterbalanced across participants.

Encoding took place in a single 15 min block. Participants were instructed to look carefully at each image and indicate whether it contained a building or not. They were not informed that their memory would be tested at a later date. Each image was presented for 2500 ms before a response screen was displayed showing “(1) Building, (2) No building”. After participants responded with the corresponding keypress, an inter-trial interval (ITI) of 1000 ms elapsed before the start of the next trial. The 160 images were presented in a randomized order.

Retrieval tested the recognition of 160 old images randomly intermixed with 80 new images. Images were presented above a five-point confidence scale: “(1) Definitely did not see, (2) Probably did not see, (3) Unsure, (4) Probably saw, (5) Definitely saw”. Participants were asked to indicate whether they remembered each image from the previous session. The trial was terminated following a response, or after a 5000 ms time limit had elapsed, and was followed by a 1000 ms ITI. Images that were incorrectly judged to contain buildings or not were excluded from subsequent retrieval analysis, since these trials suggest that images were not adequately attended to. Consistent with prior work,^6^ analysis focused on two outcome measures: (1) confidence ratings of 4 (probably saw) and 5 (definitely saw) to old images were classed as “hits”, (2) confidence ratings of 4 and 5 to new images were “false alarms”. To correct for response bias toward old/new responses, the signal detection measure A′ was calculated using hits and false alarms, for which 0.5 represents chance performance.

### Factual knowledge task—pretest

This was performed prior to learning in order to probe knowledge of the species to be learned. This involved four stages: (1) Picture Identification: participants were shown a single image and identified the animal shown from two options. This was repeated for each species. (2) General knowledge: 20 two-alternative forced choice questions regarding general characteristics of amphibians. (3) Specific knowledge: 20 two-alternative forced choice questions about information that they would encounter during the learning session, similar to questions encountered in the main test after learning. (4) Subjective disgust: participants rated the amount of disgust they felt toward each species (1 = no disgust, 9 = extreme disgust), to control for the influence of emotion on memory. (5) Subjective knowledge: participants rated their prior knowledge of each species (1 = no knowledge, 9 = extensive knowledge). All tests were self-paced and trials were presented in a random order.

### Factual knowledge task—encoding

Participants were informed that all information they were about to learn would be tested. They were shown example test questions using a species not featured in the learning and instructed to not discuss or peruse information about frogs/amphibians outside of the specified learning blocks.

Participants learned about three frogs (Poison Dart Frog, Flying Frog, Gray Tree Frog), 3 toads (Burrowing Toad, Yellow-bellied Toad, Cane Toad), three newts (Alpine Newt, Orange-bellied Newt, Great Crested Newt) and 3 salamanders (Giant Salamander, Green Salamander, Mud Puppy) in separate 30 min blocks. Characteristics (e.g., habitat) were adapted from their actual biology and behaviors.

Each day, participants learned 2 blocks in the morning (e.g., frogs and newts) separated by a 2 min break and 2 blocks containing different animal types in the afternoon (e.g., toads and salamanders). Participants learned the same species in the morning and afternoon on each of the 3 days of the experiment, but the order that they learned them was switched each day. For example, if they learned frogs and then newts on the first morning of the experiment, they learned newts followed by frogs on the second morning. Animal type was counterbalanced across participants for morning/afternoon sessions.

The learning materials for each type of animal (e.g., newts) contained roughly 80 slides of factual information in the form of numbered points and images. Participants moved forward and backward through slides at their own pace, but were advised to observe a minimum speed to ensure all slides were seen. A time counter was visible throughout and slides included markers informing how much time should have passed at 5 min intervals.

To assist learning, some slides asked participants to write on paper what they could recall. Participants were permitted to make notes which were removed at the end of each block. The final slide of each block instructed participants to use the remaining time to recap the information.

When the 30 min of each block had elapsed, participants completed a Karolinska Sleepiness Scale (KSS) and were asked 3 questions to rate on a 7-point scale: “Was your attention focused on the task or something unrelated to the task? (1 = completely on task, 7 = completely off task)”, “How motivated were you to learn the information? (1 = completely motivated, 7 = completely un-motivated)”, and “How well do you feel you could learn the information? (1 = extremely well, 7 = extremely poorly)”. These are referred to as subjective “focus”, “motivation”, and “ability”, respectively. Scores were subsequently inverted for analysis so that higher values represent higher levels for each measure.

### Factual knowledge task—retrieval

This involved two-alternative forced choice questions followed by a confidence rating (“certain”, “somewhat certain”, “guess”) (Fig. 2a). Questions and confidence ratings were displayed until a response was made (or maximum of 10,000 ms elapsed). There were 360 questions (90 for each animal type) relating to materials encoded in the morning (180) and afternoon (180) and presented randomly within 6 blocks that were separated by 30 s breaks. The foil was the answer to the same question for a different species. Participants were instructed to think carefully about each question within the time available.

Memory scores were calculated for “certain”, “somewhat certain”, and “guess” responses separately by subtracting incorrect from correct responses. “Overall memory” consisted of all correct responses, including trials where no certainty response was recorded.

### Psychomotor vigilance task

Participants performed three test batteries daily that included a 10 min psychomotor vigilance task (PVT) beginning at 10:15, 16:30, and 20:15. A counter appeared on screen at random intervals between 2000–10,000 ms, and participants responded with the space bar as quickly as possible. Failure to respond within 10,000 ms elicited an alerting tone. Lapses (responses >500 ms) were measured. All tasks were presented with E-Prime 2.0 (Psychology Software Tools, Pittsburgh, PA).

### Procedure

The experiment was conducted over 15 days as part of the Need for Sleep 4 study. Participants adhered to a 9 h sleep schedule (23:00–08:00) for one week prior to the study. They were afforded two baseline nights (B_1_–B_2_) of 9 h nocturnal TIB (23:00–08:00) before a two-cycle simulation of a typical school week in Singapore (Fig. 1)—6.5 h TIB during weekday nights and extended sleep during the weekend. The first cycle consisted of five nights of restricted sleep (SR1_1_–SR1_5_) followed by two nights of 9 h recovery sleep (R1_1_–R1_2_). The second cycle included three nights of sleep restriction (SR2_1_–SR2_3_) and two recovery nights (R2_1_–R2_2_). During sleep restriction nights in both cycles, the continuous sleep group were afforded 6.5 h nocturnal TIB (00:15–06:45 h), while the split sleep group were permitted 5 h TIB at night (01:00–06:00) and a 1.5 h afternoon nap opportunity (14:00–15:30).

All cognitive tasks were administered to participants via individual laptops in a classroom. Picture encoding took place on SR1_5_ at 16:45, with retrieval on R1_2_ at 16:45. The factual knowledge task began in the second week on SR2_1_. Participants performed the pretest prior to the first morning learning block (11:00) and returned later for the first afternoon learning block (16:45). This was repeated on SR2_2_ and SR2_3_. The factual knowledge retrieval test took place on R2_1_ at 20:30. Participants also performed 3 PVTs on each day of the experiment (10:15, 16:30, and 20:15).

### Polysomnography

Sleep was recorded using portable SOMNOtouch PSG devices (SOMNOmedics, GmbH, Germany), during selected sleep and nap episodes. EEG was recorded from two main channels (C3 and C4 according to the 10–20 system) referenced to contralateral mastoids. The common ground and reference electrode were placed at Fpz and Cz. Left and right electromyogram and electrooculogram were also attached. Impedance <10 KΩ was verified at each electrode. The sampling rate was 256 Hz. Data was scored according to standard criteria^48^ utilizing the Z3 score automated EEG scoring system^49^ and verified by a trained researcher. Sleep architecture was compared across the whole protocol, while main analyses focused on the duration of each sleep stage and TST averaged across nocturnal and nap episodes in temporal proximity to each task. Thus, for picture encoding this focused on SR1_5_, and for factual knowledge this included the mean of SR2_1_ and SR2_3_.

### Statistical analysis

Separate 2 × 2 mixed ANOVA with the factors group (split sleep/continuous sleep) and time (morning, afternoon) were performed for each measure of the factual knowledge task (certain, somewhat certain, guess, and overall memory), as well as subjective measures during learning (KSS, focus, motivation, and ability) and PVT lapses. Independent and paired *t*-tests were used for sleep architecture, picture encoding metrics, and follow-up tests to ANOVA. One sample *t*-tests compared performance on several measures to chance. Spearman’s Rho correlations explored the relationship between memory and other measures. Effect sizes indicated by Cohen’s *d* for *t*-tests of key group comparisons. All statistical tests were two-tailed, significance level *p* < 0.05. All means are presented in the text ± standard deviation.

## Supplementary information

Supplementary Material

## Data Availability

Data are available on the Open Science Framework at osf.io/6wkh2.

## Supplementary information

**Supplementary information** accompanies the paper on the *npj Science of Learning* website (10.1038/s41539-019-0047-z).
