# Supplementary material for: Does splitting sleep improve long-term memory in chronically sleep deprived adolescents?
Source: NPJ Sci Learn. 2019 Jun 28;4:8. doi: 10.1038/s41539-019-0047-z (PMC6599038; doi:10.1038/s41539-019-0047-z)
Supplement: Supplementary file 1 — Supplementary Material [file 41539_2019_47_MOESM1_ESM.pdf]

Does splitting sleep improve long-term memory in chronically sleep deprived adolescents?

James N. Cousins[a], Elaine van Rijn[a], Ju Lynn Ong[a], Kian F. Wong[a], & Michael W. L. Chee[a]

[a]Centre for Cognitive Neuroscience, Duke-NUS Medical School, Singapore, 169857, Singapore

Supplementary Table 1. Sleep macrostructure of nap periods for the split sleep group.

	N1 (min)		N2 (min)		SWS (min)		REM (min)		TST (min)	
	Mean	SD	Mean	SD	Mean	SD	Mean	SD	Mean	SD
SR1-1	1.8	1.8	31.2	12.0	27.8	13.0	10.1	10.2	70.9	15.7
SR1-3	2.0	1.4	36.7	12.3	31.0	9.4	9.8	8.4	79.5	3.9
SR1-5	3.2	4.3	32.4	10.0	29.8	12.5	13.7	10.0	79.1	6.1
SR2-1	2.7	3.5	32.8	9.8	32.8	14.1	9.8	9.4	78.1	5.3
SR2-3	2.0	2.0	33.1	9.2	31.3	12.6	12.9	10.9	79.4	5.7

Note. N1 = stage 1; N2 = stage 2; SWS = slow wave sleep; REM = rapid eye movement sleep; TST = total sleep time; SD = standard deviation
